# Neuroscience needs evolution

**DOI:** 10.1098/rstb.2020.0518

**Published:** 2022-02-14

**Authors:** Paul Cisek, Benjamin Y. Hayden

**Affiliations:** ^1^ Department of Neuroscience, University of Montréal, 2960 chemin de la tour, local 1107 Montréal, QC H3T 1J4 Canada; ^2^ Department of Neuroscience, Department of Biomedical Engineering, and Center for Magnetic Resonance Research, University of Minnesota, Minneapolis, MN 55455, USA

**Keywords:** psychology, ontology, phylogenetic history, evolutionary neuroscience, developmental neuroscience

## Abstract

The nervous system is a product of evolution. That is, it was constructed through a long series of modifications, within the strong constraints of heredity, and continuously subjected to intense selection pressures. As a result, the organization and functions of the brain are shaped by its history. We believe that this fact, underappreciated in contemporary systems neuroscience, offers an invaluable aid for helping us resolve the brain's mysteries. Indeed, we think that the consideration of evolutionary history ought to take its place alongside other intellectual tools used to understand the brain, such as behavioural experiments, studies of anatomical structure and functional characterization based on recordings of neural activity. In this introduction, we argue for the importance of evolution by highlighting specific examples of ways that evolutionary theory can enhance neuroscience. The rest of the theme issue elaborates this point, emphasizing the conservative nature of neural evolution, the important consequences of specific transitions that occurred in our history, and the ways in which considerations of evolution can shed light on issues ranging from specific mechanisms to fundamental principles of brain organization.

This article is part of the theme issue ‘Systems neuroscience through the lens of evolutionary theory’.

## 1. Introduction

Like all biological entities, the brain and nervous system are products of evolution. That is, they were produced slowly, over millions of years, through a long series of modifications yielding a diversity of forms that were pruned by natural selection. Of course, almost all scientists agree with this. Evolution is the ultimate explanation for both the fundamental principles and the specific details of biological systems at all levels, and it can be considered the ‘grand unifying theory’ of biology [1]. Nonetheless, while evolution is effectively unanimously accepted among scientists, we believe its implications for neuroscience have not been nearly as recognized as they should be. Indeed, we believe a great deal of neuroscience research, especially at the systems level, proceeds almost as if it doesn't take evolution into account. For the most part, this work does not reject evolutionary principles, but rather it fails to consider just how much those principles can guide our research. In other words, much of systems neuroscience is neglecting to use one of the major tools available to it.

To some extent, this neglect of evolution is due to the influence of various disciplines that gave rise to modern systems neuroscience. In particular, psychology was originally explicitly conceived as the study of human mental processes in separation from questions of biology [2]. Likewise, cognitive science, a major influence on much of modern systems neuroscience, was originally conceived as a study of the ‘software’ of the mind as opposed to the ‘hardware’ of the brain [3,4]. While modern cognitive and systems neuroscience seeks to connect across levels, the fundamental concepts (e.g. ‘attention’, ‘working memory’, etc.) are still those outlined by psychological traditions. Consequently, the mechanisms of animal behaviour are often interpreted in terms of theories designed to explain human cognition, as if evolutionary history and diversity are irrelevant.

Indeed, the neglect of evolution also reflects the fact that most of neuroscience is oriented towards understanding the human brain. Even ostensibly basic, curiosity-driven research is typically funded by agencies with a mandate to develop treatments for human diseases. While basic systems neuroscience makes widespread use of animal models, they are generally just that—models for the human brain—and are valued to the extent that they have external validity to humans. As a consequence, features that differentiate the brains of these animals are under-appreciated and under-studied. The result is a science focused on one specific species as if it were an end in itself rather than a stepping stone in a long evolutionary process.

Another possible reason for the limited influence of evolutionary principles on systems neuroscience is that their importance is unfamiliar. Thus, one goal of this introduction is to describe, with several examples drawn from the literature, how evolutionary thinking can guide neuroscience. Indeed, that is also the goal of this entire theme issue.

## 2. The importance of evolutionary history

The concept of natural selection is often used in systems neuroscience, but when it is used, it is most often employed simply as the justification for assuming the optimality of neural systems with respect to some performance criteria. That is, evolution is treated as if it was nature's process for finding optimal solutions to specific problems posed by the world. Tacit within this view is the assumption that the solutions arrived at by natural selection are independent of the process of evolution and can be understood without it. When put plainly like this, however, it is clearly a false assumption. Evolution has no goal, no metric that defines something as a specific problem, aside from the general problem of differential survival. Instead, it produces variations of an ancestral system and then, through natural selection, favours those variations that happen to accomplish something useful, sometimes overcoming what *used to be* a problem. It is not directed toward any goal, neither optimality nor complexity, but simply biases the expansion of diversity. Because it has such massive resources and so much time, evolution can eventually accomplish amazing things, but it is not a process for finding solutions to any specific computational or cognitive problems we might define.

Nevertheless, one could still argue that the mechanisms produced by the evolutionary process are independent of that process and can be understood without it. This argument is plausible at face value, but it ignores that other pillar of evolutionary theory: descent with modification. That is, for a variation to enter into the game of natural selection, it must first be *possible*, and the range of possible variations is highly constrained by the ancestral system. There are many reasons why the space of possible mechanisms is more constrained than one might imagine on the basis of purely functional concerns, such as optimality. In particular, in multi-cellular organisms, a major source of constraint comes from the developmental process. The genome does not describe a blueprint for the body or a connectome for the brain; it describes a recipe for constructing the body and brain through a long series of developmental changes. As in any recipe, later stages depend on earlier ones, so modifications cannot be introduced arbitrarily. Modifications to early stages are unlikely to be adaptive if only because they will usually violate the assumptions under which later stages operate. For this reason, successful new variations of species tend to be those that add new stages at the end of development, duplicate systems that then specialize differently, or abandon entire chains of developmental sequences and regress. Consequently, the evolution of animals along any lineage is highly conservative, and as a result, the structures and mechanisms of modern animals are strongly dependent on the structures and mechanisms of their ancestors and cannot be understood outside that ancestral context.

What are the implications for neuroscience? As neuroscientists, we are faced with a black box—the brain—whose complexity is daunting. However, we have at our disposal an invaluable crutch. That black box did not pop into existence all at once, but was constructed slowly, over time, without any deliberate designer. Furthermore, the process of its construction was not arbitrary but took place within extreme constraints, which guided its development as much or more so than optimality. Finally, and most importantly, there exist approaches that can estimate the steps of that process with reasonable confidence, and a great body of research that has already been doing so for decades, providing insights that can help us to answer questions about structure and function that are otherwise incomprehensible [5]. It seems unwise for us to ignore such insights and to explore a vastly under-constrained space of theories, most of which are simply not compatible with the brain's biological reality.

**Figure 1.**
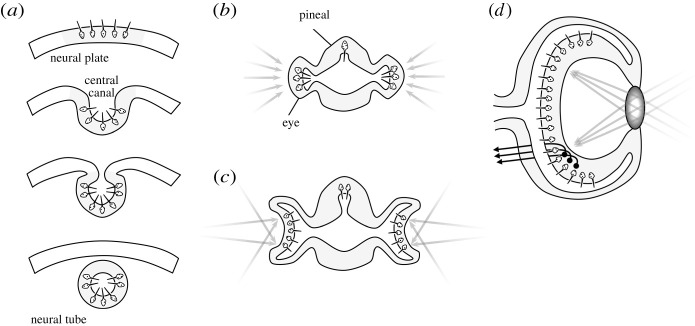
The history of the vertebrate eye [6]. (*a*) The photosensitive cells of the eumetazoan apical nervous system (top) folded inward with the formation of the neural tube in chordates (bottom), such that their ciliated receptors pointed into the central canal. (*b*) In cephalates, the rostral neural tube bulged laterally to form the eyes and dorsally to form the pineal gland. (*c*) Next, the lateral eyes folded inward into cups, such that different parts of the sensory surface now became exposed to different parts of the world, conferring spatial topography. (*d*) The vertebrate eye folded further inward, and a lens formed over the aperture, allowing the projection of focused images onto the retinal surface.

One famous example of the severe constraints imposed by evolutionary history is the vertebrate retina, which, as every first-year neuroscience student knows, is organized backwards. That is, the primary sensory cells face away from the lens, such that light has to pass through layers of horizontal, bipolar, amacrine and ganglion cells before striking the photosensitive receptors. What could be the adaptive reason for this? Many candidate explanations have been proposed, from filtering to protection, but the real answer is much simpler—the answer is that there *isn't* anything adaptive about it. The inverted organization of the retina is simply a consequence of the evolutionary history of the eye [6]. As shown in figure 1*a*, the photoreceptors originally pointed outward from the neural plate, but then folded inward when the neural tube was formed. This was not a problem for our ancient chordate ancestors, which were almost completely transparent and only used their vision to modulate circadian rhythms and occasionally escape from a passing shadow. But since then, the photoreceptors have been trapped in that inward-pointing orientation, and natural selection could not simply flip them around without wrecking the rest of the developmental recipe for establishing visual circuitry. When parts of the neural tube migrated outward to form the lateral eyes (figure 1*b*), the path of least resistance was to just keep the non-receptive layers transparent. Thus, the inverted retina has been retained, even in some of the world's most visually impressive animals like cats, hawks and humans. By contrast, in cephalopods, which do not undergo neurulation, the retinal receptors face outward. The vertebrate retina is an example that ‘nothing in biology makes sense except in the light of evolution’, as Dobzhansky [5] famously said, and similar examples abound in all fields of biology, including neuroscience.

Another example of the importance of history in neuroscience arises in the context of research into the functional neuroanatomy of memory. The traditional view of memory holds that there is a single episodic memory system in the brain and evidence suggests it is situated in the medial temporal lobe [7]. Murray *et al*. [8] argue that while this view is plausible, it does not readily accord with what we know about the evolutionary history of primates. Indeed, they argue that evolutionary considerations are sufficient to cast serious doubt on the traditional view and instead make use of what we know about the evolution of primates, of learning systems, and of the brain, to suggest an alternative view.

They propose that we have seven different memory systems, which evolved at different times, and serve distinct roles. These systems include (1) reinforcement memory, (2) navigation memory, (3) biased competition memory, (4) manual foraging memory, (5) feature memory, (6) goal memory and (7) social subjective memory. This categorization of memory is different from the more traditional one based on first principles and that categorizes memory by its general properties; instead, this is driven by evolutionary understanding. Indeed, each form of memory is associated with a specific time in evolutionary history. For example, (1) reinforcement memory evolved first along with control of movement and (2) navigation memory arose later as early creatures could not just move locally but began to navigate. At the tail end, (7) our social memory systems evolved in the human lineage, that is, within the past 5 million years. Notably, these seven forms of memory correspond to distinct brain substrates: for example, (2) navigation memory is associated with the hippocampal complex while (3) biased competition memory is associated with agranular prefrontal cortex. Of course, these forms of memory do not necessarily correspond neatly to single brain areas—for example, they suggest that (7) social memory is associated with granular prefrontal regions.

Murray and colleagues' arguments about memory systems illustrate an important point that transcends the neuroscience of memory. Specifically, they show that evolutionary history—even when our knowledge of it is incomplete—provides an important source of information that can guide our search for answers to questions in neuroscience. And, notably, this help is not limited to peripheral systems, such as the retina and the early visual system. Evolution can help us to understand even higher-level cognition.

Just as evolution can provide important information about microscale circuitry and macroscale functional anatomy, it can also provide guidance in the understanding of behaviour [9,10]. One notable example comes from the neuroscience and psychology of decision-making [11,12]. This is, of course, a prominent topic in the field of neuroscience. Often, our decisions are influenced or even determined by the values of the options available to us, and the typical way of approaching how we make such choices is based on economic principles. The standard approach—again, derived from first principles and not from evolution—holds that the simplest and most fundamental decision problem is a choice between two simultaneously presented options; the rest of the science of decision-making is then an extension of this. According to this view, the brain computes the subjective utility of those options using a single value scale (a ‘common currency’), chooses the one with the larger magnitude on that scale, and turns that choice into an action plan [13–15].

However, while this view is the foundation of a great deal of theoretical and empirical work, it is inconsistent with the principles of foraging theory [16]. Foraging theory is a branch of behavioural ecology that was inspired by microeconomics but was shaped by considerations of biology and the principles of evolution [17]. Importantly, in real natural situations, animals rarely happen to encounter two offers, simultaneously, among which they can only select one. Instead, choice options tend to appear in isolation. When the single option appears, the decision-maker must either accept or reject it and move on. Thus, unlike economic theory, foraging theory is based on the foundation of an accept–reject decision.

Kacelnik *et al*. [11] have artfully shown that mental models of choice based on accept–reject elements are very different from those based on two-option choices. Even in ostensibly binary choice, decision-makers set an internal threshold based on their estimate of the richness of the environment and then evaluate the first option they encounter as an accept–reject decision. They then move on to contemplate the second option, having used the information about the value of the first one to update their threshold, and proceed to contemplate the options, in sequence, until they select one. These principles provide a ready explanation for ostensibly aberrant behavioural patterns [18] and motivate neural models that emphasize the serial contemplation of options, even in an ostensible simultaneous choice paradigm [19–21]. They also help explain the successes of choice models that emphasize the role of affordances, and competition between them, in determining the outcomes of choice processes [22]. These types of choice models tend to be more robust and have promise for understanding behaviour and neural activity in continuous choice contexts, rather than in the arguably less naturalistic discrete contexts studied in the laboratory [23].

Indeed, we believe that the guidance of evolutionary principles is strong enough that they may cause us to alter basic organization of psychological concepts. That is, an evolutionary perspective can even help us reconsider the questions we ask about the brain [24], doing so in a manner that respects its biological nature and is not burdened by preconceptions of philosophical history. As noted above, much of the conceptual foundation for systems neuroscience comes from psychology, which defined the elements of mental processes in terms of concepts such as ‘memory’, ‘attention’, ‘perception’, etc.—terms that were useful to describe human mental life. The goal of systems neuroscience is often described as finding the neural mechanisms that implement these processes, but this cannot succeed if they are erroneously defined. For example, while introspection suggests that our perceptions are filtered through something we call ‘attention’, a great deal of research questions whether attention corresponds to a real entity or distinct system in the brain [25–29]. Instead, a look at evolutionary history suggests that certain phenomena associated with attention (visual selective attention) are more closely related to decision-making [30]. This encourages collaboration between research groups that traditionally studied these concepts independently, using different methods and paradigms. That is, evolution helps us not only to find new methods and insights for finding answers to our questions, but to define better questions to ask in the first place.

## 3. Systems neuroscience through the lens of evolutionary theory

The examples above highlight the potential for evolutionary principles to provide insights that have utility for neuroscience, whether they be on the relationship between function and anatomy, or whether they be on the way we think about behaviour. Such examples serve two functions. First, they illustrate the general point that evolution has much to offer for neuroscience. Second, they provide specific cases for evolution that can be used to answer follow-up questions. For this reason, we proposed this theme issue of *Philosophical Transactions B* designed to include these cases—articles by scholars interested in applying evolutionary insights to solving neuroscience problems.

These scholars do not identify as a group, nor do they feel that their research is all within the same subfield (nor is the list exhaustive). Instead, these researchers come from a broad assortment of subfields, but are united by their commitment to taking evolution seriously. Consequently, the goal of this theme issue is to highlight how considerations of evolutionary history can shed light on questions in systems neuroscience. We believe that, considered together, these papers both offer a strong argument in favour of using evolutionary principles and serve as a series of examples. Moreover, the specific ideas in these papers can serve as a jumping-off point for future investigations. As such, we believe this issue can serve as an example of a different style of systems neuroscience, one that more fully respects the brain's biological nature than traditional approaches.

Vertebrate evolution has been remarkably conservative, and a powerful demonstration of just how conservative it has been is provided in the paper by Shreyas Suryanarayana, Brita Robertson, and Sten Grillner [31]. These authors summarize and synthesize an impressive body of work, using some of the most advanced techniques in neural recording, anatomical tracing, and electrical and optical stimulation, to show that the basic outline of the vertebrate brain is similar between mammals and lamprey, a species whose lineage diverged from ours about half a billion years ago. This includes fundamental brainstem and spinal mechanisms of locomotor control [32], midbrain mechanisms of steering, approach and escape responses [33,34], as well as forebrain circuits such as the basal ganglia [35] as well as olfactory [36], somatosensory and visual systems in the pallium [37], which are proposed to be homologous with piriform and neocortical systems in mammals. The implication is profound: that all of these basic circuits existed in our last common ancestor and thus, have been around for hundreds of millions of years. This is important, of course, because theories that assign functions to these regions need to take account of their past to be plausible. This does not mean that these regions did not adapt and alter their function, but they could do so only within the constraints of their history.

Even deeper homologies are discussed by Thurston Lacalli, whose paper [38] summarizes work on the amphioxus, an invertebrate chordate whose ancestors diverged from ours more than 600 million years ago [39] and remained in their filter-feeding niche for most of that time [40]. It possesses many of the hypothalamic and locomotor control systems of vertebrates as well as homologues of some visual pathways, though it lacks complex sense organs and has only a vague precursor of a telencephalon. Lacalli proposes that despite its humble brain and behaviour, the amphioxus may provide a window into large conceptual and philosophical questions, such as how sensory consciousness first emerged among mobile animals. This work, distant as it may seem from the daily concerns of neuroscientists who work with mice or monkeys, provides an invaluable comparison point, showing both what tends to be preserved and what tends not to be, over long evolutionary timescales. Indeed, we believe that familiarity with a broad range of species and their nervous systems, especially ones that are phylogenetically distant from humans, is a valuable resource for active neuroscientists [41–43], even ones ostensibly only interested in the human or primate brain.

## 4. The importance of major transitions in our evolutionary past

Evolution may occur slowly, but it can have a great effect. Across generations, it can produce large alterations with corresponding adaptations. In addition to papers that emphasize the conservative nature of evolution, our issue includes work that describes several major transitions that took place along the human lineage and made us what we are today. One significant example is the transition from an aquatic to a terrestrial environment. Malcolm MacIver & Barbara Finlay [44] discuss what this meant for sensory systems, especially vision. Due to the properties of light diffraction in water versus air, upon getting out on land our ancestors encountered a visual world that expanded dramatically, by a factor of a million in terms of sensed volume [45]. This offered a vast expansion of opportunities for navigation, as well as decision-making and planning. But there were also new challenges, such as the need for multi-joint limbs and the circuitry to control their movement and posture. All of this produced a great deal of neural expansion and diversification, leading to specific innovations that we find in extant animals, including ourselves.

Other implications of the water to land transition are discussed by Lucia Jacobs, whose paper proposes how air-breathing set the stage for hippocampal evolution in terrestrial tetrapods [46]. Her olfactory navigation hypothesis [47] suggests that olfaction is not just about odour identification, but fundamentally about using odours for spatial navigation. When our ancestors emerged onto land, olfactory sampling became linked with respiration, and Jacobs proposes that this can explain hippocampal theta rhythms, how they could be used to keep track of distance and ultimately for scaffolding mammalian memory.

Another dramatic transition started with the mammalian retreat into nocturnal life and then, about 200 million years later, a return to diurnal life in some primate species. This is described in a paper by Jon Kaas, Hui-Xin Qi and Iwona Stepniewska, which focuses on the corresponding changes to the visual system [48]. In particular, unlike other mammals, primates evolved good vision even when still nocturnal. This was made possible by their large, frontally facing eyes, as well as by a shift in the balance of visual projections to the neocortex, reducing the pathway through the superior colliculus and expanding the more direct retino-geniculo-striate pathway. This was followed by an expansion of the dorsal stream of visual processing into a wide variety of action-specific domains in parietal and premotor regions.

The paper by Paul Cisek summarizes many of these transitions, following along our lineage from chordate filter feeders to mobile aquatic vertebrates, terrestrial tetrapods, nocturnal mammals and diurnal primates [49]. Instead of framing the associated neural innovations as the superposition of new circuits at increasing levels of a hierarchy, with primate cognition at the top, he describes them as the progressive elongation of a general feedback control circuit that gradually subdivided into finer and finer control systems. That is, the highest level of the control hierarchy is the most ancient ‘hypothalamic’ regulation of behavioural state, within which new subdivisions such as abstract planning appeared as adaptations that extended control further into the world and toward more abstract interactions. The resulting architecture, he suggests, retains an ancestral organization into parallel control systems dedicated to guiding particular species-typical actions. Selection between these systems is governed by the basal ganglia, while a selection of specific actions within the chosen system occurs through a competition within each specific cortical map.

Giovanni Pezzulo, Thomas Parr and Karl Friston echo some of these points, emphasizing feedback control as the fundamental organization of the nervous system, but extend it with predictive processing [50]. In particular, they emphasize that predictive processing is by no means a recent evolutionary innovation, but rather a basic principle of vertebrate neural organization that was elaborated from allostatic control to multiple sensorimotor loops that extend in terms of both spatial hierarchy and temporal scales. In this view, cognitive abilities are not added as a new system on top of an old sensorimotor controller, but rather emerge as an extension that specializes part of it toward increasingly abstract and long-term control.

A different but compatible perspective is offered by David Leopold and Bruno Averbeck, who discuss how the vertebrate brain trains itself, a process they refer to as ‘self-tuition’ [51]. They propose that hypothalamic systems modulate telencephalic systems to bias them toward learning the types of information needed for basic functions such as feeding, seeking mates and escaping from threats, as well as orienting and navigating around the world. The complexity of the primate brain, they propose, reflects the complexity of such interactions.

A still more general theoretical treatment of similar issues is offered by Stuart Wilson and Tony Prescott, who define a mathematical framework for how layered control architectures operating at different temporal scales can coordinate to produce complex behaviour [52]. Importantly, while it is widely acknowledged that slower processes can provide the constraints on faster ones, these authors show how the inverse can also be true. The result is a control architecture without a strict hierarchy, but where different levels mutually constrain each other.

## 5. The importance of evolution for understanding ourselves

These and other insights from considering our evolutionary history provide important clues for understanding the particular idiosyncrasies of the primate brain. A key theme is ‘embodiment’, or the proposal that much of brain function is ultimately aimed at controlling our interactions with the world. Of course, it could not be otherwise—from the perspective of natural selection, even the most sophisticated abilities to passively contemplate existence are of no consequence if they don't translate to some behavioural outcome that affects survival. As Thomas Huxley said, ‘the great end of life is not knowledge, but action’. This motivates us to consider even the most abstract cognitive abilities of humans and other animals as serving a role in interaction, colouring how we might think of their neural mechanisms. For example, Justin Fine and Benjamin Hayden propose that the entire prefrontal cortex of anthropoid primates, long considered to be the seat of abstract cognition, can be reconsidered as an extension of premotor circuits for movement control [53]. In particular, they note the continuity of anatomical and physiological gradients spanning both the lateral and medial aspects of the frontal lobe and the computational similarity of mechanisms for selecting movements or abstract goals. Ultimately, they propose that regions often considered as specialized for representing economic choice variables can be seen as a hierarchically organized system serving a broader role—the selection of motor goals. They particularly focus on the orbitofrontal cortex (OFC), long associated with valuation and comparison, and show that it is equally if not better understood as the starting point for a prefrontal hierarchy that selects goals and guides the implementation of actions (see also [54]).

Along similar lines, Louise Barrett, Peter Henzi and Robert Barton suggest that our understanding of primate social behaviour is best framed in terms of their interactive behaviour, extending the functional architecture of action control and selection toward more abstract domains of interaction [55]. For example, they discuss how social coordination demands sophisticated control of one's position in space with respect to others, and how this may have evolved as an elaboration of more ancient mechanisms of affordance-based action guidance. They suggest that the demands of these kinds of ‘embodied’ concerns set the stage within which primate social cognition evolved, and that they are a better framework for understanding it than traditional frameworks emphasizing abstract knowledge and meta-representation of other minds.

Such an attitude of reframing old questions can be taken even further. As Luiz Pessoa, Loreta Medina and Ester Desfilis suggest, it can be used to reconsider many aspects of human psychology [56]. These authors point out that many of the basic theoretical distinctions used to discuss mental phenomena (e.g. attention, emotion, etc.) come from pre-scientific ideas about the mind, and do not necessarily fit with what we find when we look at the brain. For example, they argue that while perception makes convenient sense as a term to describe some of what the brain does, in implementation, perception is not markedly separated from processes like cognition and action. In other words, the categories used in cognitive psychology do not readily carve nature at the joints. They suggest, in agreement with others [24,57], that a more promising basis for delineating the functional architecture of the brain is to step away from traditional mental categories and toward the regulation of complex behaviours that drove brain evolution during its long history.

The paper by Joseph LeDoux makes a related point, in the context of consciousness and especially the experience of emotional states [58]. He points out that one should not assume consciousness to be a single thing, with a single explanation, but rather a preliminary term for a set of phenomena that can be distinguished in terms of their evolutionary history. In particular, the primary response to threatening stimuli is clearly an ancient property of many animals, but the subjective experience of fear is something quite different, and possibly much more recent to our lineage. Generalizing outside the specific case of fear, LeDoux suggests that the kinds of conscious feelings that characterize human emotions arise from cognitive interpretations of the significant situations we encounter in life.

In addition to the theoretical perspectives and proposals discussed in many of the papers in this issue, some also address specific datasets and important methodological issues. For example, the paper by Margaret Bryer and colleagues demonstrates through phylogenetic analysis how a specific behavioural capacity is distributed across a range of species and what that tells us about its history [59]. They take the case of sensitivity to numerical quantities, showing that species differ systematically in their ability to engage in numerical cognition. Remarkably, they then extend this idea to link numerical cognition abilities to a specific aspect of brain morphology, neuronal density. This work is unusual in its broad species perspective—by comparing 48 different species, it has a rare vantage point in terms of breadth that lets it draw firmer conclusions than other studies with a narrower focus on one or a few species. In particular, the use of a large number of species lets them draw conclusions about the evolution of cognition, something that is notoriously hard to draw inferences about.

Comparison of behavioural capacities across species, however, is itself difficult. Any two species might solve a given task through different mechanisms that both produce similar behaviour; conversely, two similar mechanisms may behave differently simply because one species finds the way a task is presented to be unnatural (e.g. a visual discrimination task presented to a rodent versus a primate). A. David Redish and colleagues propose that one method for translating across species is to consider the computations that each performs [60]. That is, some methods establish the validity of a task from one context to another, or one species to another. They propose that one can likewise operationalize behavioural processes as a computational algorithm, which has its own form of validity. One example would be an integration of information that produces a rise to threshold process. Such ‘computational validity’ can then be used for very concrete goals, such as identifying which insights from studies of rodents are most likely to translate to viable treatments of mental disorders in humans. They propose that this form of validity can stand next to other forms, like external validity and face validity, and can therefore serve a useful purpose in allowing us to draw inferences from model organisms, even ones that are otherwise quite different from us.

The reader may note that in this theme issue, we have focused primarily on research questions pertinent to the lineage that ultimately produced humans, and do not include work on the specific innovations of insects, molluscs and other protostomes. That is because the lineage that leads to these animals diverged from ours so long ago that most of their evolutionary story is very different from ours and has its own twists and turns that would motivate a second theme issue. We also focus more on animals that remained in a similar niche after diverging from our lineage, such as lizards, as opposed to those that dramatically changed their lifestyles, such as birds. This is simply a strategy for limiting the scope and length, and not in any way meant to downplay the valuable insights provided by such studies. And indeed, studies of birds are central to the paper by Bryer *et al*. demonstrating an excellent example of convergent evolution [59].

## 6. Conclusion

A typical graduate program in neuroscience will include a series of courses that serve as the intellectual foundations of the field. These will often include, for example, courses in neuroanatomy, in molecular biology, development and physiology. Without disputing the importance of these foundational courses, we propose that the principles of evolution are just as important. Indeed, we hope the examples given above and in the rest of this theme issue provide a strong argument that almost any reasoning in systems neuroscience ought to include consideration of evolutionary history. For example, students should learn about evolutionary principles beyond natural selection, including the importance of developmental changes, the broad diversity in brain types, the different ways that brains can solve common problems, and the actual evolutionary history of the brains they seek to understand.

Ultimately, we believe that evolutionary history is important enough to neuroscience that our incomplete understanding of the deep biological past is a limiting factor on our ability to make sense of the brain. As such, we believe that basic research into evolutionary history is critical for advancing the goals of neuroscience. The insights it provides complement insights from anatomy, physiology and behaviour, and should be part of the basic foundation of the knowledge we bring to bear upon our goals for understanding the brain.

## Data accessibility

This article has no additional data.
